# A review on gut microbiota and migraine severity: a complex relationship

**DOI:** 10.1007/s10787-025-02023-2

**Published:** 2025-11-27

**Authors:** Noha M. Gamil, Rana M. Ghorab, Reham Z. Elsadawy, Nada M. Khadrawy, Mohamed Abdelhamid, Khalid A. Ismael, Omar A. Mohamed, Mohamed M. Ata, Habiba T. Jalal, Joumana E. Zeidan, Reem T. Rashed, Riham A. El-Shiekh

**Affiliations:** 1 Department of Pharmacology and Toxicology, College of Pharmaceutical Sciences and Drug Manufacturing, Misr University for Science and Technology (MUST), P.O. Box 77, Giza, Egypt; 2 College of Pharmaceutical Sciences and Drug Manufacturing, Misr University for Science and Technology (MUST), P.O. Box 77, Giza, Egypt; 3 Department of Pharmacognosy, Faculty of Pharmacy, Cairo University, Kasr El-Aini Street, Cairo, 11562 Egypt

**Keywords:** Dietary triggers, Gut microbiota, Inflammatory mediators, Novel treatments, Migraine-gut axis, Probiotics, Short-chain fatty acids, Neurotransmitters

## Abstract

The gut-brain axis plays a vital role in migraine pathophysiology. Studies highlight reciprocal interactions between the central nervous system and the gastrointestinal tract. Previous research suggests that factors such as gut microbiota profiles, inflammatory mediators, neuropeptides, serotonin pathways, stress hormones, and nutritional substances influence this interaction. The pathophysiology of migraine has been linked to changes in the gut-brain axis, which affects migraine severity and frequency. Additionally, dietary approaches, including the ketogenic diet, vitamin D supplementation, omega-3 intake, probiotics, and weight loss plans, have shown promising effects in reducing migraine symptoms by positively impacting the gut microbiota and the gut-brain axis. Understanding these connections could lead to novel therapeutic strategies for effectively managing migraines. It is worth noting that research highlights several innovative treatments for migraine, such as Zelirex and Cevimide, implantable devices like Cefaly and Revilion, and new effective routes of administration for Sumatriptan. Finally, patients’ perspectives and concerns were thoroughly discussed, with a focus on future directions in the migraine-gut axis research.

## Introduction

Migraines are one of the most debilitating and troublesome disorders. The prevalence of migraine, a recurring primary headache condition, is 18.9% in women and 11.4% in men (Huang and Xu 2020; Christensen et al. 2021). The condition imposes a significant economic burden, with annual healthcare costs in the U.S. estimated at $20 billion due to lost productivity and medical expenses (Christensen et al. 2021). Migraine significantly impacts both physical and mental health, as it can make it difficult to function at work or school, which significantly reduces quality of life and heightens social isolation (Orr et al. 2016). The issue becomes much more significant when other comorbidities, including autoimmune, gastrointestinal (GI), and psychiatric disorders, are considered. While the pathophysiological process underlying migraine is not yet fully understood, emerging research highlights the role of the GI system in migraines (Doulberis et al. 2017; Buse et al. 2020).

However, here’s a brief illustration of the relationship between the gut and brain axis. The phrase “gut-brain axis” indicates two-way communication between the central nervous system (CNS) and the gastrointestinal system. The brain generally controls movements and the functioning of the GI system (secretion and sensory) (Weltens et al. 2018). Hormonal variables influence gut functions by modulating stress responses through the hypothalamic-pituitary-adrenal (HPA) axis. It is believed that the gastrointestinal system can also impact the central nervous system, where the gut affects numerous brain functions, including behaviour, cognition, and even nociception (Pires et al. 2024).

Numerous neurological conditions, including multiple sclerosis, mood and anxiety disorders, Alzheimer’s disease, Parkinson’s disease, and migraines, have been associated with dysfunction of the gut-brain axis. Additionally, various neurotransmitters such as gamma-aminobutyric acid, dopamine, serotonin, and calcitonin gene-related peptide (CGRP) are believed to be involved in this process. Gut microbes can communicate with the central nervous system through at least three interconnected routes: immunological, endocrine, and nervous system signalling pathways (Arzani et al. 2020; Aurora et al. 2021; Sgro et al. 2023). Gut dysbiosis also affects extra-neural pathways: (1) gut-liver axis—microbiota-derived LPS worsens hepatic inflammation, raising CRP and fibrinogen associated with migraine chronification (Bui et al. 2023; Naghipour et al. 2023; Vakilpour et al. 2024) (2) gut-heart axis—SCFAs modulate autonomic tone, reducing the risk of cardiac arrhythmia in migraine sufferers (Amini-Salehi et al. 2024).

Therefore, the discovery that GI symptoms such as nausea, vomiting, and gastroparesis are clinical indicators of migraine highlights the potential link between the two systems. Additionally, the condition known as abdominal migraine, which presents with both abdominal and migraine symptoms, suggests that both affected systems may share a common cause. Furthermore, gastrointestinal disorders (GID) such as irritable bowel syndrome (IBS), celiac disease (CD), inflammatory bowel disease (IBD), and Helicobacter pylori (H. pylori) infection (HPI) often co-occur with migraine (Galli et al. 2021).

Shared inflammatory pathways, such as TNF-α and IL-6 upregulation, and gut dysbiosis-driven serotonin depletion are involved in both GI disorders and migraine, indicating a bidirectional relationship mediated by systemic inflammation (Galli et al. 2021). To summarise, the pathophysiology of over 25 CNS diseases has been linked to the GI tract (GIT) microbiota. Various mechanisms have been proposed to explain this association, including bacterial translocation resulting from an impaired intestinal barrier, immune cell migration, and the systemic diffusion of microbial products or metabolites (Doulberis et al. 2017; Yin et al. 2022; San Mauro Martín et al. 2024).

This study presents new evidence of a two-way interaction between the gut-brain axis and migraine treatment. It highlights the potential of dietary interventions and non-pharmacological therapies, such as the ketogenic diet, vitamin D supplementation, and probiotics, alongside conventional medications. By introducing new drugs like Zelirex and Cevimide and developing innovative delivery systems for existing medications, this research supports a more comprehensive approach to migraine management. Ultimately, it lays the foundation for ongoing research aimed at improving the lives of those affected by migraines.

## Methods

We conducted a thorough review of recent studies focusing on factors such as gut microbiota, inflammatory mediators, neuropeptides, serotonin pathways, stress hormones, and dietary components affecting migraines. To maintain a systematic approach, we set clear inclusion and exclusion criteria for selecting studies. We included studies published in the last 10 years that involved humans and provided quantitative data on the link between gut microbiota and migraine severity. We excluded studies with insufficient data, those not published in English. We also evaluated various nutritional interventions, such as the ketogenic diet, vitamin D supplementation, higher omega-3 intake, probiotics, and weight loss efforts, for their potential to reduce migraine symptoms. Additionally, emerging treatments like medications Zelirex and Cevimide, devices such as Cefaly and Revilion, and new methods for administering sumatriptan were considered.

## Results

Our results indicate a strong connection between the gut-brain axis and the frequency and severity of migraines. Dietary approaches that support a healthy gut microbiome appear to help in lowering migraine symptoms. New pharmaceutical and device-based therapies have shown promising results in migraine management. Patient feedback emphasises the need for personalised treatment plans and identifies important directions for future migraine research.

## Discussion

### Anatomy and physiology of the two interconnected systems

There is an overlap in the pathophysiology of both migraine and GI comorbidities, including disorders of gut-brain interaction (DGBI). However, neither of these complex conditions has had its full pathogenesis understood. Exploring this overlap may offer insights into a shared underlying biological process or abnormality. Evidence now suggests that GI issues and migraines share similar pathophysiological mechanisms (Aurora et al. 2021). This common pathophysiology is thought to arise from the interplay of various factors such as gut microbiota, neuropeptides, inflammatory mediators, and the serotonin pathway. Since migraine and gastrointestinal dysfunction exhibit similar symptoms like nausea, vomiting, dyspepsia, and gastroparesis, it is believed that the autonomic nervous system (ANS) plays a role in linking these two conditions (Cámara-Lemarroy et al. 2016). Typically, the enteric nervous system, Cajal interstitial cells, fibroblast-like cells, stomach smooth muscle, the central nervous system, and the autonomic nervous system work together in a bidirectional way to control digestion. The nucleus tractus solitarius (NTS) in the brainstem receives sensory signals from the GI tract via the vagus nerve (Mawe et al. 2023). Efferent sympathetic or parasympathetic nerves influence GI function by altering secretion and movement. In disease states, gastroparesis, or delayed stomach emptying, can occur due to damage or loss of Cajal interstitial cells. This damage may result from oxidative stress and immune dysregulation driven by macrophages. Sometimes, fibrosis in the muscular layers and loss of enteric nerves also contribute to this condition (Aurora et al. 2021).

**Fig. 1:**
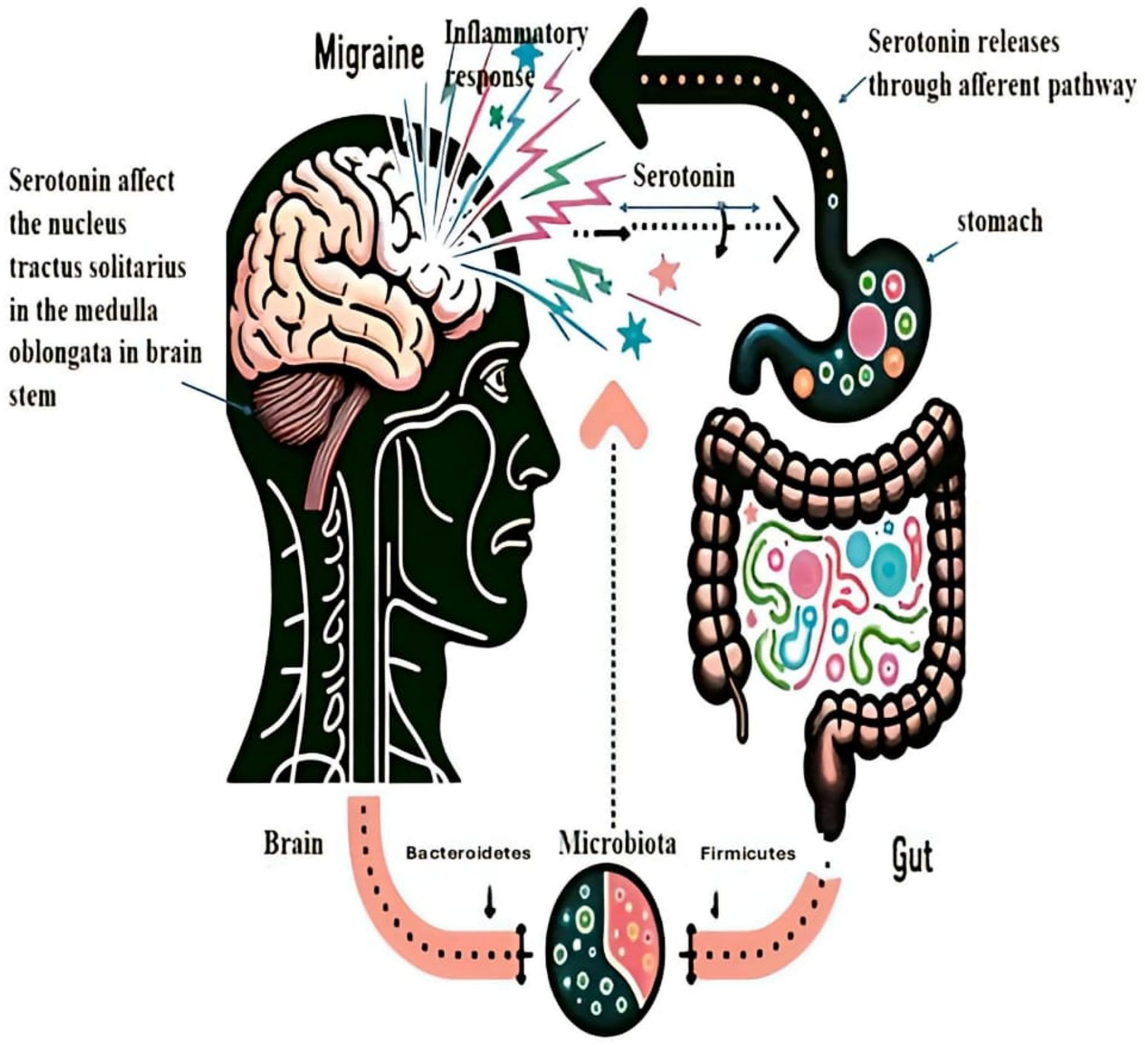
The relationship between gut microbiota and migraine severity

Migraine symptoms include delayed gastric emptying, nausea, and vomiting. Activation of the trigeminovascular (TGV) system during a migraine is believed to stimulate neuronal activity in the NTS, which controls nausea and vomiting (Spekker et al. 2021). Serotonergic signaling influences migraine pathology, especially regarding nausea, vomiting, and gastric emptying. Serotonin acts as both a vasodilator and vasoconstrictor, regulating pain sensation. It is proposed that reduced activity of 5-HT1B/1D receptors triggers the TGV system, which may initiate migraine episodes (Socała et al. 2021) (Fig. 1).

95% of the body’s total serotonin is located in the gut, and multiple studies with serotonergic drugs indicate that serotonin might play a role in regulating GI dysfunction symptoms and gastric emptying (Wei et al. 2021; Mori et al. 2022). Stimulation of 5-HT4 receptors enhances neurotransmitter release in reflex pathways, while serotonin activates 5-HT1P receptors to trigger peristaltic and secretory reflexes. Activation of 5-HT3 receptors on visceral afferent fibers can induce vomiting, whereas inhibiting these receptors may slow intestinal movement. Additionally, 5-HT3 receptors on extrinsic sensory nerves help transmit information from the stomach to the central nervous system. Inflammation also leads to decreased serotonergic signalling in the mucosa (Tack et al. 2009; Aurora et al. 2021).

Research on 5-HT1A agonists shows they improve stomach accommodation, reduce post-meal symptoms, and alleviate common gastroparesis issues like nausea and vomiting in patients with gastroparesis and functional dyspepsia. Besides the serotonergic receptors mentioned earlier, migraine mechanisms have also been associated with other receptor subtypes in the GI tract, including dopaminergic and adrenergic receptors (Aurora et al. 2021).

The enteric nervous system (ENS), along with the sympathetic and parasympathetic branches of the autonomic nervous system (ANS), as well as neuroimmune and neuroendocrine signaling pathways, comprise the complex communication network between the gut and the CNS (Fig. 1). Visceral feedback from the intestines is conveyed by afferent spinal and vagal sensory nerves to the spinal cord (thoracic and upper lumbar regions) and the nucleus of the solitary tract. These nerves interact with polysynaptic pathways that connect to higher brain regions, such as the limbic forebrain and hypothalamus. Brain regions influencing vagal and spinal autonomic output to the viscera include the cingulate and insular cortex, amygdala, bed nucleus of the stria terminalis, and hypothalamus (Duan et al. 2021). The gut-brain axis is regulated in both directions by autonomous neuronal projections from these systems. Additionally, hormones and humoral signaling molecules are involved alongside the neuronal pathways previously mentioned (Margolis et al. 2021).

Additionally, migraine is linked to gastrointestinal conditions like IBS or inflammatory bowel disease (Cámara-Lemarroy et al. 2016). Moreover, a significant difference exists in the prevalence of Helicobacter pylori infection between migraine sufferers and controls (44.97% vs 33.26%). Moreover, abdominal migraine is now recognised as a disorder of the gut-brain axis and is classified among pediatric functional abdominal pain conditions (Socała et al. 2021).

**Fig. 2:**
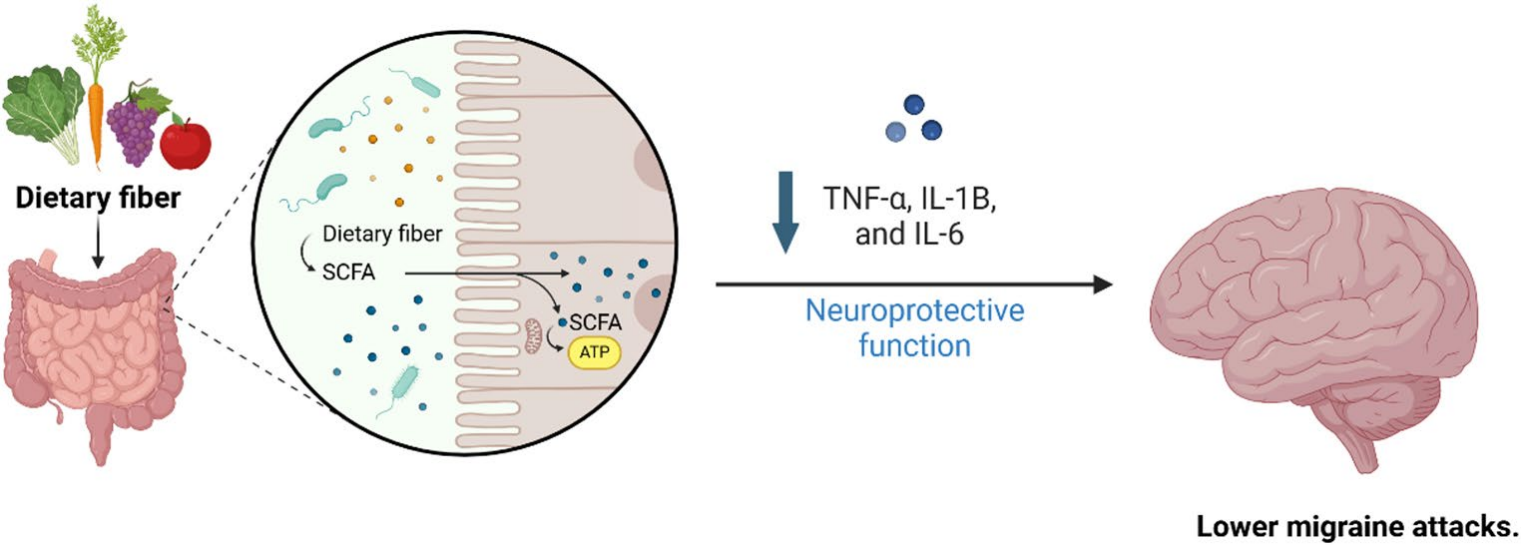
Impact of diet on gut microbiota and neuroinflammation

Leaky gut can lead to increased levels of proinflammatory cytokines like TNF-α, IL-1β, and IL-6, potentially affecting nociceptive responses in the trigeminal pathway and contributing to the initiation of migraine pain (Vuralli et al. 2024b). In wild-type (WT) mice, antibiotic therapy was shown to extend the duration of nitroglycerin (NTG)-induced acute migraine-like pain. This prolongation was completely avoided if TNF-α was genetically deleted or if a TNF-α receptor antagonist was injected into the intra-spinal trigeminal nucleus caudalis (Sp5C). Additionally, antibiotic treatment increased the NTG-induced up-regulation of TNF-α in the Sp5C (Tang et al. 2019). Probiotic administration significantly lessened the prolongation of migraine-like pain caused by antibiotics. Moreover, NTG-induced migraine pain was notably greater in GF mice compared to WT mice, and colonising the gut with faecal microbiota from WT mice effectively reversed the increased pain associated with microbiota depletion. Collectively, these results suggest that gut microbiota dysbiosis elevates TNF-α levels in the trigeminal nociceptive system, contributing to the persistence of pain similar to migraine (Talafi Noghani and Namdar 2019) (Fig. 2).

For migraine sufferers, taking a probiotic pill may help speed up gastric emptying and decrease gastric stasis. However, methodological differences prevented a meta-analysis of randomized placebo-controlled studies on probiotics for migraine prevention, according to PRISMA guidelines (Parohan et al. 2020). A qualitative review of the trials produced mixed results: the second experiment indicated highly significant benefits, whereas the first showed no notable difference in migraine frequency or intensity. Although the study’s statistical analysis (paired t-test within groups) did not reveal a change in migraine frequency after probiotic treatment, it has still faced criticism (Talandashti et al. 2024). A recent study, not included in the systematic review, found that the average frequency of migraines decreased following the use of symbiotics (Talafi Noghani and Namdar 2019).

Strong evidence connects gastrointestinal disorders with migraines (Welander et al. 2021). Conventional wisdom suggests that headaches may originate from the gastrointestinal (GI) tract through neurovascular pathways. The GI tract can influence the trigeminovascular system (TVS) and induce neurogenic inflammation via neuronal connections (NTS-TNC) and systemic circulation (histamine). Thus, when addressing headache issues, it is crucial to pay close attention to any disruptions in the GI tract (Aurora et al. 2021).

### The role of gut microbiota in migraine pathophysiology

The gut microbiota, mainly consisting of Bacteroidetes and Firmicutes, the two most dominant bacterial groups in healthy individuals, influences the host via neural, immune, and metabolic mechanisms. The connections between the enteric microbiota and the brain include the vagal nerve, tryptophan metabolites, and microbial products like short-chain fatty acids (SCFAs) (Nicholson et al. 2012). Gut microbiota influence brain function by regulating neurotransmission systems like serotonergic, noradrenergic, dopaminergic, glutamatergic, and GABAergic, either affecting neurotransmitter synthesis or producing them directly (Table 1)(Chen et al. 2021).

Certain bacteria synthesise serotonin, such as *Escherichia coli*,* Enterococcus*, and *Streptococcus*. *Bifidobacteria* and *Lactobacillus* produce GABA, while *Lactobacillus* also generates acetylcholine. *Bacillus* is responsible for producing dopamine, and *Escherichia* produces norepinephrine (Talafi Noghani and Namdar 2019). Except for GABA, which is more likely to cross the blood-brain barrier (BBB) due to the presence of GABA transporters, it is unlikely that other neurotransmitters produced in the gut will reach the brain. However, by affecting the ENS, neurotransmitters generated in the stomach may indirectly influence the brain. Furthermore, tryptophan metabolic pathways leading to derivatives such as serotonin, kynurenine, or indole are regulated by enzymes present in the gut flora (Fung et al. 2017). Bacteria influence brain serotonin levels by modulating tryptophan, a serotonin precursor. The microbiota impacts the concentration of serotonin in the brain. It is well established that the gut-brain axis is considerably affected by the composition of gut bacteria (Dinan and Cryan 2017).

**Table 1:** Production of neurotransmitters by gut microbial species and their influence on migraine and Gastrointestinal functions

Gut Microbe	Neurotransmitter(s) Produced	Role in Migraine and GI Function	References
*Lactobacillus*	GABA, Acetylcholine	GABA influences CNS functions and neuroinflammation, which is relevant to migraine management, while acetylcholine is involved in gut motility.	Zhao et al. (2022)
*Bifidobacterium*	GABA, Serotonin	GABA helps reduce pain pathways, while serotonin influences mood and gastrointestinal motility. Their balance is crucial for managing migraines.	Duranti et al. (2020)
*Escherichia coli*	Serotonin, Norepinephrine	Serotonin influences gastric motility and nociceptive pathways, whereas norepinephrine affects the autonomic nervous system.	Mavros et al. (2024)
*Enterococcus*	Serotonin	Serotonin influences gastrointestinal motility and migraines by affecting neuroinflammation and regulating serotonin levels both centrally in the brain and peripherally.	Legan et al. (2022)
*Streptococcus*	Serotonin	Serotonin production influences gastrointestinal function and migraine severity.	Arzani et al. (2020)
*Bacillus*	Dopamine	Dopamine influences brain activity, mood, and pain pathways, contributing to migraines.	Hamamah et al. (2022)
*Ruminococcus gnavus*	Unknown	Increased gut permeability can boost neuroinflammation, contributing to migraine pathophysiology.	Rehman et al. (2022)

**Fig. 3:**
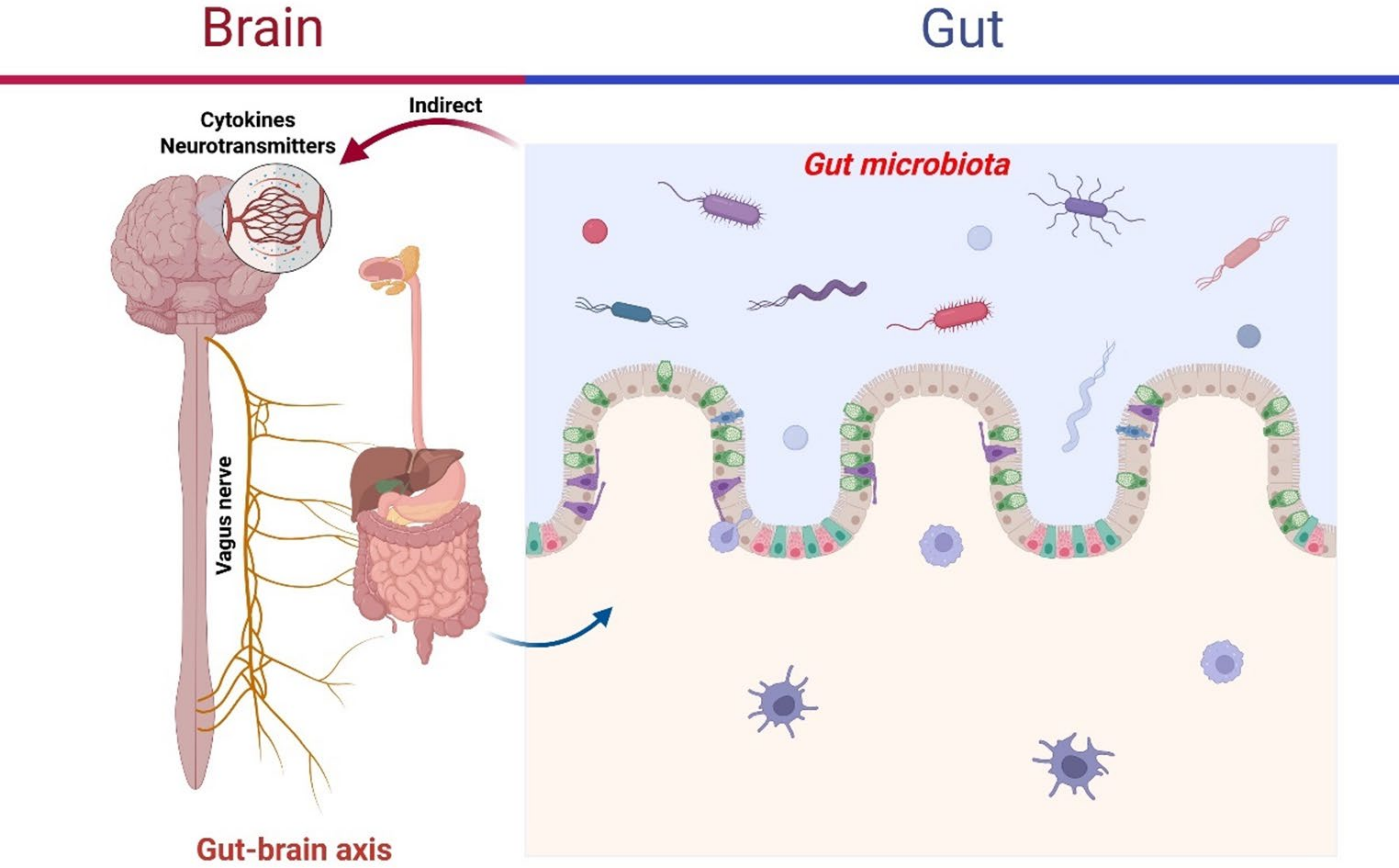
Direct and indirect communication channels between the gut microbiota and CNS

This process involves two methods (Fig. 3): indirect signalling, impacting hormones, inflammatory chemicals, and neurotransmitters from the microbiota; and direct connecting, which stimulates vagus nerve end terminals. Since the CNS can regulate the gut microbiota via the sympathetic and parasympathetic nervous systems and the release of neuroendocrine peptides, this mechanism operates bidirectionally (Holzer and Farzi 2014).

Physical and psychological stress factors can impact on the gut microbiome. These stressors trigger the hypothalamus to release corticotropin-releasing hormone, which prompts the adrenal glands to produce cortisol. This hormone can influence intestinal permeability and alter the microbiota profile, potentially leading to dysbiosis—disruptions in the gut microbiota. Conversely, dysbiosis and heightened gut permeability can activate the HPA axis by releasing proinflammatory cytokines like IL-1β and TNF-α. While the stress-induced steroid response may suppress cytokine production, it can also increase the risk of inflammatory disorders (Petrella et al. 2014).

Furthermore, multiple gut bacterial strains are believed to be vulnerable to the antimicrobial effects of CGRP, substance P (SP), vasoactive intestinal peptide (VIP), and neuropeptide Y (NPY), suggesting their involvement in the bidirectional communication between the gut and the brain. It has also been observed that SP levels in the colon increase following antibiotic treatment and the associated dysbiosis. However, administering Lactobacillus piracies appears to reduce this impact (Holzer and Farzi 2014).

Research on animal models indicates that peripherally administered CGRP can decrease both basal and stimulated gastric acid secretion (Christensen et al. 2021). Moreover, CGRP strongly inhibits pancreatic enzyme secretion by influencing central vagal outflow. However, it’s important to note that microbiota may impact CGRP signaling (Wang et al. 2022). Migraine sufferers show lower levels of *Bifidobacterium longum*, which has anti-inflammatory properties, and higher levels of *Ruminococcus gnavus*; which is pro-inflammatory (Talafi Noghani and Namdar 2019). *B. longum* suppresses TNF-α and IL-6 through SCFA production, while *R. gnavus* worsens gut permeability, increasing trigeminal nociception (Cohen et al. 2022). TNF-α boosts CGRP release from trigeminal neurons, increasing neurogenic inflammation. Meanwhile, gut microbiota-derived serotonin affects CGRP expression through 5-HT1B/1D receptors, connecting dysbiosis to migraine pathways (Cohen et al. 2022).

Dietary factors play a crucial role in supporting the gut microbiome’s ability to sustain immune function and overall gut health. In the distal colon, bacteria produce short-chain fatty acids (SCFAs) such as butyrate, propionate, and acetoacetate. These vital fatty acids are essential for preserving the integrity of the gut barrier (Park and Floch 2007). Probiotics and fiber are dietary elements that influence gut microbiota activity and the resulting SCFA levels in the gut. These SCFAs can circulate through the bloodstream and reach the CNS, where they exhibit neuroprotective properties. For example, sodium butyrate boosts the production of glial-derived neurotrophic factor (GDNF) and brain-derived neurotrophic factor (BDNF). It also encourages cell differentiation and proliferation in the dentate gyrus (Yoo et al. 2011). Furthermore, butyrate decreases TNF-α production in the brain by inhibiting histone deacetylases, leading to reduced NF-κB activation and lower levels of pro-inflammatory cytokines. This process helps mitigate neuroinflammation in the trigeminal pathway, which is a major factor in migraine pain. Changes in the SCFA-producing bacterial phyla can also influence the host’s immune system (Mennigen and Bruewer 2009).

Dietary strategies, such as adding fermentable fibers (prebiotics) to a high-fat diet, can restore depleted butyrate-producing bacteria and Bifidobacteria, emphasising the key role of diet in shaping gut microbiota composition (Park and Floch 2007). The bidirectional link between gut permeability and inflammation indicates that LPS leakage due to increased gut permeability can trigger inflammatory and immune responses, which may further enhance gut permeability. Conversely, proinflammatory cytokines like TNF-α, IL-1β, and IL-6 can influence nociceptive responses in the trigeminal pathway and contribute to migraine onset (Mennigen and Bruewer 2009).

### Pharmacological gut-targeted treatments for migraine

Traditional pharmacological gut-targeted therapies for migraine have focused on managing these GI symptoms and improving drug absorption, as well as exploring the gut-brain axis as a therapeutic target. Key approaches include the use of antiemetics and prokinetic agents (e.g., metoclopramide, domperidone), triptans, and nonsteroidal anti-inflammatory drugs (NSAIDs), often in combination to optimize both headache relief and GI comfort (Ashina et al. 2021; Yang et al. 2025).

- *Triptans*

Triptans, particularly sumatriptan, represent a pivotal class of traditional pharmacological agents that demonstrate significant gut-targeted effects in migraine management. While primarily recognised as 5-HT1B/1D/1F receptor agonists targeting the trigeminovascular system, triptans exhibit important interactions with gastrointestinal function that underscore their role in gut-brain axis modulation (Martin et al. 2018; Aurora et al. 2021; Ebogo-Belobo et al. 2022).

Sumatriptan’s relationship with gastrointestinal function is multifaceted, addressing both the complications arising from migraine-associated gastroparesis and the drug’s own absorption challenges (Fuseau et al. 2001). During migraine attacks, gastroparesis frequently occurs, leading to delayed gastric emptying that significantly compromises the absorption and effectiveness of oral antimigraine medications. This gastroparesis may arise during or between migraine attacks and appears to decrease the consistency, absorption, and efficiency of oral treatments, creating a therapeutic paradox where the condition requiring treatment simultaneously impairs treatment delivery (Assadpour et al. 2022).

The oral bioavailability of sumatriptan is notably low at approximately 15% due to extensive hepatic first-pass metabolism and incomplete gastrointestinal absorption. This pharmacokinetic limitation has prompted the development of alternative delivery routes that bypass gastrointestinal complications while potentially providing superior therapeutic outcomes. Intranasal formulations, particularly the dry nasal powder system (AVP-825), demonstrate reduced treatment-emergent nausea compared to oral tablets and maintain absorption independent of gastrointestinal system function (Assadpour et al. 2022).

Clinical studies have demonstrated that sumatriptan exhibits antiemetic properties that extend beyond its primary antimigraine effects, providing therapeutic benefits for the nausea and vomiting commonly associated with migraine attacks. This antiemetic action occurs through multiple mechanisms, including modulation of serotonin pathways in both the central nervous system and enteric nervous system. The drug’s ability to cross the blood-brain barrier, as demonstrated in recent regional distribution studies, suggests that sumatriptan may exert central effects on gut-brain axis signaling that contribute to its overall therapeutic profile (Svane et al. 2024).

Emerging research has revealed that triptans influence tryptophan metabolism pathways that are crucial for gut-brain axis communication. Multi-omic analyses of triptan-treated migraine attacks have provided insights into molecular mechanisms involving glutamine metabolism, cAMP regulation, and fatty acid oxidation, all of which are intimately connected to gastrointestinal function and metabolic signaling between the gut and brain. These findings suggest that triptans’ therapeutic effects may extend beyond their established vascular and neuronal targets to include metabolic modulation of gut-brain axis dysfunction (Kogelman et al. 2023).

Pediatric research has further illuminated the connection between triptans and gut-brain axis function, demonstrating that tryptophan metabolites and gut microbiota play important roles in migraine pathogenesis and potentially in triptan effectiveness. This relationship suggests that the therapeutic benefits of triptans may partially depend on their ability to modulate gut microbiota-derived metabolites that influence brain function through the gut-brain axis (Tack et al. 1998; Sakamoto 2012; Wei et al. 2021; Liu et al. 2024).

- *NSAIDs*

Nonsteroidal anti-inflammatory drugs represent another cornerstone of traditional pharmacological gut-targeted migraine therapy, though their mechanisms involve complex interactions between anti-inflammatory effects and gastrointestinal consequences. NSAIDs provide therapeutic benefits in migraine through multiple pathways that intersect with gut-brain axis function, including modulation of neuroinflammation, prostaglandin synthesis inhibition, and effects on intestinal barrier function (Pardutz and Schoenen 2010; Ozdarska et al. 2025).

The anti-inflammatory properties of NSAIDs directly address the neuroinflammatory component of migraine pathophysiology. This involves interactions between neurons, glial cells, vascular structures, and immune pathways, with critical roles played by pro-inflammatory cytokines that can originate from or be influenced by gastrointestinal inflammation. NSAIDs’ ability to inhibit cyclooxygenase enzymes and reduce inflammatory mediator production provides therapeutic benefits that extend beyond simple analgesic effects to include modulation of gut-derived inflammatory signals (Ozdarska et al. 2025).

However, the relationship between NSAIDs and gastrointestinal function in migraine patients presents significant clinical complexities. Chronic NSAID use, widespread in medication overuse headache (MOH), has been associated with intestinal hyperpermeability and “leaky gut” syndrome, which paradoxically may exacerbate the gut-brain axis dysfunction that contributes to migraine pathophysiology (Vuralli et al. 2024a). Clinical studies have demonstrated that chronic migraine patients with NSAID overuse headache exhibit elevated lipopolysaccharide levels, inflammatory molecules, and markers of intestinal barrier dysfunction in systemic circulation (Vuralli et al. 2024a). Research in medication overuse headache models has revealed that chronic NSAID administration leads to increased serum levels of lipopolysaccharide-binding protein (LBP) and occludin, indicating disrupted intestinal barrier function. This disruption allows bacterial endotoxins to enter systemic circulation, promoting low-grade inflammation and elevated pro-inflammatory molecules such as HMGB1, IL-6, and IL-17, which may contribute to central sensitization and maintenance of chronic headache patterns (Vuralli et al. 2024a).

Despite these complications, selective COX-2 inhibitors such as celecoxib have demonstrated potential advantages in migraine treatment by providing anti-inflammatory effects with reduced gastrointestinal toxicity compared to non-selective NSAIDs. Low-dose celecoxib formulations provide acute migraine analgesia with fewer associated cardiovascular and gastrointestinal events, suggesting that targeted anti-inflammatory approaches may optimize the therapeutic benefits while minimizing gut-related adverse effects (Ailani et al. 2023).

The interaction between NSAIDs and gut microbiota represents another important consideration in their use as gut-targeted migraine therapies. Research has demonstrated that NSAIDs can significantly alter gut microbiota composition, promoting the overgrowth of certain bacterial species while suppressing others. These microbiota alterations may influence the production of metabolites that affect gut-brain axis signaling, potentially modulating migraine susceptibility and treatment response (Hodkovicova et al. 2022; Zádori et al. 2023).

Clinical studies in chronic migraine patients with medication overuse headache have revealed specific microbiota alterations that correlate with inflammatory serum parameters and migraine food triggers. The dysbiosis observed in these patients appears to favor inflammatory bacterial populations, creating a feedback loop where NSAID-induced gut changes may perpetuate the inflammatory milieu that contributes to chronic migraine maintenance (Vuralli et al. 2024a).

Ibuprofen is one of the most studied NSAIDs for migraine, with evidence showing dose-dependent efficacy and gastrointestinal effects. A Cochrane review of nine studies with 4373 participants found 400 mg more effective than 200 mg, especially for 2-hour pain relief and headache relief, with better outcomes at 400 mg. Soluble forms like liquigel show faster early efficacy, beneficial for gastroparesis-related absorption issues, though standard forms catch up by 2 h (Rabbie et al. 2010). Gastrointestinal side effects are mixed: more abdominal pain with 400 mg but less nausea, possibly due to symptom relief. A meta-analysis of 137 randomised controlled trials with 89,445 participants confirmed ibuprofen’s role as a first-line treatment option, particularly when considering its favourable benefit-risk profile and widespread accessibility (Karlsson et al. 2024) .

Naproxen is a well-established NSAID with unique pharmacokinetics suitable for migraine, especially with gastroprotective strategies (Law et al. 2011). Its longer half-life offers sustained relief for prolonged attacks. A Cochrane review found moderate efficacy, with an NNT of 11 for pain-free response at 2 h, suggesting limited clinical utility as monotherapy. Real-world studies show it outperforms other non-opioid analgesics like ibuprofen (Ruscheweyh et al. 2023). Its longer action requires gastroprotective measures, especially in patients with GI issues (Targownik and Thomson 2006; Suthisisang et al. 2010). Guidelines favour naproxen’s cardiovascular safety over other NSAIDs, making it preferred for patients with low GI but high cardiovascular risk. For high GI risk patients, combining with proton pump inhibitors is advised (Scarpignato et al. 2015).

Ketorolac, a potent NSAID, is effective for severe migraine treatment, comparable to opioids, especially when administered parenterally (Puledda et al. 2024). It shows superior efficacy over some alternatives and can bypass gastrointestinal issues, making it valuable for patients with gastroparesis or vomiting (Nurathirah et al. 2022; Guan et al. 2024). Intranasal formulations with lidocaine (ROX-828) ensure safety and efficacy (Pfaffenrath et al. 2012). Pediatric studies comparing intranasal versus intravenous ketorolac found non-inferiority for pain reduction, suggesting that needle-free administration maintains therapeutic benefits while potentially reducing treatment-related distress (Tsze et al. 2022). Used frequently in emergency settings, ketorolac provides rapid pain relief, often with antiemetics, outperforming oral options due to its quick onset (Cisewski and Motov 2019). However, its gastrointestinal and renal risks require cautious limiting of ketorolac use to short-term administration (≤ 5 days). The drug’s mechanism of action, through potent cyclooxygenase inhibition, provides significant anti-inflammatory effects that may benefit gut-brain axis dysfunction, but this same mechanism underlies its enhanced gastrointestinal toxicity profile (Nurathirah et al. 2022).

Diclofenac has potent anti-inflammatory properties that may reduce systemic inflammatory mediators that contribute to both gastrointestinal dysfunction and migraine pathophysiology, representing a dual-targeting approach to gut-brain axis modulation. Clinical evidence suggests that diclofenac has effects on cyclooxygenase-2 pathways that may be particularly relevant for patients with inflammatory bowel conditions or other gastrointestinal inflammatory states. Its availability in both oral and topical formulations allows for individualized treatment approaches based on patient tolerance and concurrent gastrointestinal disorders. Diclofenac potassium powder for oral solution has demonstrated efficacy in clinical trials for severe migraine treatment. This rapidly dissolving formulation may provide faster absorption and onset compared to standard tablet preparations. It potentially addresses gastroparesis-related absorption delays and facilitates administration in patients experiencing nausea or vomiting, as it can be consumed with minimal fluid volumes (Engel and Cheng 2020).

Celecoxib offers a newer NSAID approach for migraine, with potential gastrointestinal safety and maintained anti-inflammatory effects (Ailani et al. 2023). Its selective COX-2 inhibition aims to reduce gut toxicity compared to non-selective NSAIDs. Studies show celecoxib has similar efficacy to traditional NSAIDs but with better GI tolerability. Low-dose (200 mg daily) forms are promising for both acute and preventive use. Its safety profile makes it ideal for patients with GI risks or needing long-term therapy. COX-2 selectivity targets inflammatory pathways in the gut-brain axis without GI complications linked to COX-1, possibly enabling longer use in chronic migraine and GI disorders, though cardiovascular risks should be considered.

Combination approaches utilizing NSAIDs with triptans have shown synergistic benefits in migraine treatment, with formulations combining sumatriptan and naproxen sodium demonstrating enhanced efficacy compared to either agent alone. This synergistic action may reflect complementary mechanisms addressing different aspects of gut-brain axis dysfunction, with triptans modulating serotonergic signalling and NSAIDs reducing inflammatory processes that contribute to migraine pathophysiology (Xu et al. 2016).

The integration of NSAIDs into comprehensive migraine management strategies requires careful consideration of their dual effects on gut-brain axis function. While their anti-inflammatory properties provide important therapeutic benefits, their potential to disrupt intestinal barrier function and alter gut microbiota necessitates judicious use, particularly in patients at risk for medication overuse headache.

- *Civamide*

Civanex, civamide nasal solution, acts as a TRPV-1 receptor modulator, vanilloid receptor agonist, and neuronal calcium channel antagonist. Excitatory neurotransmitters like substance P and calcitonin gene-related peptides are released less (Saper 2002). This depletion of neurotransmitters in neurons causes a reduction in their release from the trigeminal plexus, leading to decreased vasodilation, plasma extravasation, and serotonin release in the meningeal and dural blood vessels. This process may help alleviate migraine headaches. The trigeminovascular system includes both trigeminal neuralgia and cerebral blood vessels (Rapoport 2012).

- *Zelrix™ (A Novel Transdermal Formulation of Sumatriptan)*

Zelrix delivery, the Sumatriptan iontophoretic transdermal system, may offer an effective, non-invasive alternative to oral triptan treatments for migraine sufferers. It could be particularly appealing to those experiencing gastrointestinal symptoms such as nausea and vomiting during attacks. Since nausea can deter patients from taking oral medication, this transdermal approach might encourage early treatment by providing a more tolerable delivery method. A dissolving microneedle patch containing dihydroergotamine mesylate, made from polyvinylpyrrolidone, is used to treat acute migraine episodes (Pierce et al. 2009; Goldstein et al. 2012).

Moderate to severe acute migraine sufferers might find relief through dihydroergotamine mesylate (DHE), a derivative of ergot that is especially effective for patients unresponsive to triptan derivatives. However, existing DHE formulations, like subcutaneous injections and nasal sprays, face issues such as pain, side effects, and low bioavailability. To address these problems, a new delivery method is needed that allows painless self-administration, quick action, and increased bioavailability. The team developed a dissolving microneedle patch (MNP) made from polyvinylpyrrolidone, chosen for its high-water solubility and favorable solubility characteristics. Previous research shows this MNP is painless and easy to use. (Esim et al. 2021; Dali and Shende 2023).

- *Implantable devices*

Examples of such devices include those that use electrical nerve stimulation (ENS). The pain-sensing nerves in the brain play a role in the development of migraines (Evans et al. 2023). Ongoing efforts focus on developing new therapies that can alter brain activity and block pain signal transmission. ENS targets specific nerves with a safe electrical current to help reduce and relieve migraine symptoms (Moisset et al. 2020). This treatment targets three primary nerves: the trigeminal nerve in the forehead, regulating facial movement and sensation; the vagus nerve, controlling respiration, heart rate, and digestion, connecting the brain to the abdomen; and the occipital nerve at the scalp’s rear (Lenaerts et al. 2008).

Examples of implantable devices in the treatment of migraine:

*Cefaly* is an FDA-approved over-the-counter device designed to prevent migraines. To use it, place the device on your forehead for twenty minutes daily to help prevent migraines. During a migraine, wear it for up to an hour to alleviate pain. The patient controls the intensity of the electrical impulses. Cefaly is only suitable for adults without pacemakers or any metal or electronic devices in the brain. Possible side effects include tingling or headaches (Riederer et al. 2015).

*Relivion™* works by sending electrical signals to the trigeminal nerve in your forehead and the occipital nerve at the back of your head through the skin. When worn during the start of a migraine for up to an hour, it can help control the severity (Rodríguez-Ortiz-de-Salazar et al. 2022).

### Agents being considered for clinical use in migraine prevention: emerging therapeutics and patented compounds

A new era of migraine prevention strategies has started with specific pharmacological approaches targeting the calcitonin gene-related peptide pathway and newly discovered neuropeptide systems. The very first ever pathophysiology-directed anti-migraine drugs are four FDA-approved monoclonal antibodies (mAbs) for prophylaxis: erenumab (Aimovig^®^, Amgen; US Patent 11,466,090 B2), the only CGRP receptor antagonist that is administered subcutaneously at 70–140 mg monthly with a responder rate of 43–50% in episodic migraine; galcanezumab (Emgality^®^, Eli Lilly; US Patent 11,498,959 B2) and fremanezumab (Ajovy^®^, Teva; US Patents 10,899,826 B1, 11,028,160 B2), which target the CGRP ligand with monthly or quarterly dosing who show significant reduction in monthly migraine days in EVOLVE and HALO trials; eptinezumab (Vyepti^®^, Lundbeck; US Patents 11,639,381 B2, 12,215,145 B2), the intravenous formulation that offers fast onset on day one post-infusion in PROMISE trials. Compared to standard preventive agents, these mAbs possess better safety profiles with much lower dropout rates due to adverse events (-16.2% vs topiramate) and higher sustained effectiveness over 52 weeks in observational studies.

Orally bioavailable small molecules, which are CGRP receptor antagonists (“gepants”), have been developed for use as acute and prophylactic therapeutics. The 2-hour pain freedom is witnessed in 19–21% of patients in ACHIEVE trials with ubrogepant (Ubrelvy^®^, AbbVie), formerly Allergan patent number: 12,168,004 B2, and its continuation patent application previously filed. Rimegepant (Nurtec^®^ ODT), Pfizer’s product, has received patent 11,083,724 B2 as the first dual-indication medication; it was approved to be taken (75 mg) for both attack and prevention, and it decreased monthly migraine days from 4.3 days, which was recorded for placebo users, to 3.5 days only. Atogepant (Qulipta^®^, AbbVie), the first gepant developed for chronic treatment with oral formulation delivery, has demonstrated ≥ 50% responder rates of 56–61% in episodic migraine headache (ADVANCE trial) and reduces monthly headache frequency by ≥ 6/9 days in subjects with chronic positional migraine overuse with drug-induced headache (PROGRESS trial). Zavegepant (Zavzpret^®^, Pfizer; US Patent 12,030,868 B2) also gained approval from the FDA in 2023, whereby they recommended that emergency treatment be initiated by administration of intranasal ten milligrams, which took over two hours to show improvement, remaining 23%, compared to stroking the head or taking a placebo at 15%. In addition to greater efficacy compared to triptans, gepants have certain class benefits, such as the lack of vasoconstriction, making them ideal for cardiovascular disease, and the absence of medication misuse headache impact (Rissardo and Caprara 2022; Ashina et al. 2023; Blair 2023; Lipton et al. 2025).

Migraine prevention is on the brink of a novel development in the pituitary adenylate cyclase-activating polypeptide (PACAP) class, with Lu AG09222 (Lundbeck), a humanized monoclonal antibody against PACAP, recently shown to be effective in patients with difficult-to-treat migraines. In a Phase 2 trial published in the New England Journal of Medicine (2024), the 750 mg dose demonstrated a decrease in monthly migraine days by 6.2 compared to placebo, which showed 4.2 per month (difference − 2.0 days, *P* = 0.02), with a side-effects profile similar to that of the placebo. Remarkably, PACAP38-induced migraines take place even though there is no CGRP pathway, meaning there are other ways of treating or so-called preventive medicine for this group of patients. The ongoing phase 2b/3 PROCEED trial will look at four doses and is expected to be reported in late 2025 (Ashina et al. 2024; Pellesi et al. 2024; Diener 2025; Al-Karagholi et al. 2025).

There is the advent of novel combination therapies that try to tackle the multi-factorial nature of migraine. The combination of an IGF-1R activator and a CGRP inhibitor (US Patent 12,324,827 B2) provides a dual-barreled approach to neurovascular inflammation, as well as neuronal neuroprotection. eIF4E translation initiation inhibitors (US Patent 12,329,756 B2)—cercosporamide, eFT508, and 4EGI-1—decrease CGRP-driven preclinical models and trigeminal sensitization, with potential for combination with gepants or triptans. Monoclonal antibodies to CGRP combined with onabotulinumtoxinA for the treatment of resistant forms show good results in real-world studies and are yet to be confirmed in prospective trials (Corasaniti et al. 2023).

The most current evidence suggests an individualized and gradual treatment approach, as follows: start treatment with oral gepants or anti-CGRP monoclonal antibodies (mAbs), depending on the patient’s preference, attack frequency, and comorbidities. PACAP inhibitors and combination therapies are reserved for those who do not respond to other treatments. Network meta-analyses confirmed that CGRP mAbs have better safety profiles and that gepants have dual acute/preventive action. Integration of gut-brain axis modulation and targeted pharmacotherapies, such as probiotics, dietary interventions, and CGRP-targeted agents, may provide synergistic effects that address both peripheral and central mechanisms. With the fact that the pathophysiology of migraine headache is well understood, there will be more novel targets to employ, including VIP, orexin new agents, and mitochondrial function regulators. This will further increase the therapeutic choices, making it possible to transform migraine from a complex disorder into a self-controlled disease that can be treated precisely (Damen et al. 2025; Li et al. 2025).

### Non-pharmacological approach to modulate the migraine-gut axis

**Fig. 4:**
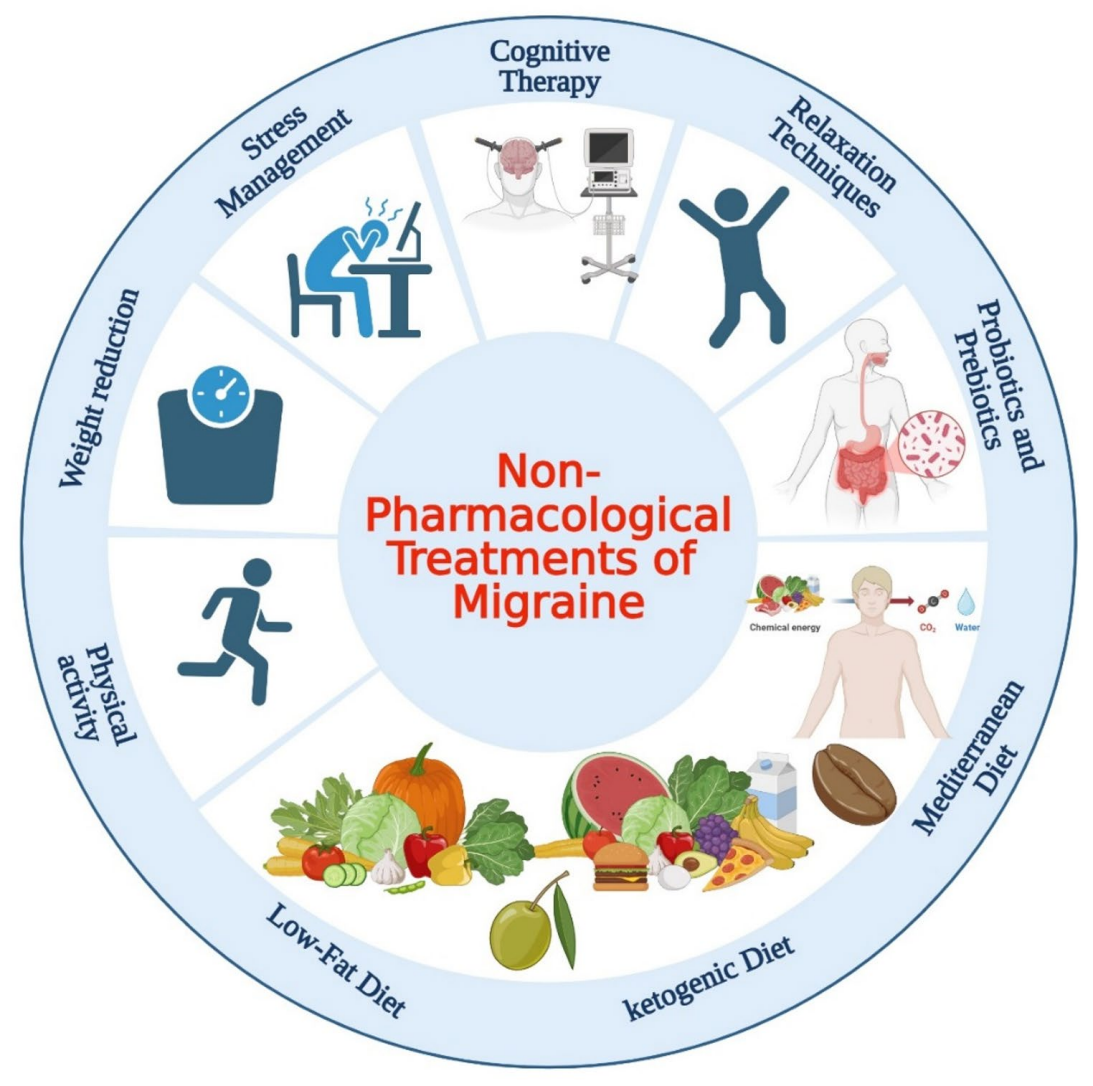
Non-pharmacological approach to modulate the migraine-gut axis

In addition to traditional pharmaceutical treatments for migraine, there is increasing attention on an adjunct therapy involving dietary changes and lifestyle adjustments (Fig. 4). These modifications have been demonstrated to beneficially affect gut microbiota, which could significantly influence migraine symptoms (Gazerani et al. 2024).

- *Physical activity*

WHO advises engaging in physical activity to enhance quality of life (Ergezen 2023). The recommended amount of physical activity is 75–150 min of vigorous aerobic exercise weekly or 150–300 min of moderate aerobic activity. Engaging in these activities can reduce the risk of depression, cardiovascular diseases, diabetes mellitus, and some types of cancer (McInnis and Morehead 2020). Physical exercise has also been associated with beneficial neurological effects, including regulation of the HPA axis, decreased inflammation, and enhanced neuroplasticity (Ignácio et al. 2019) .

Physical activity can influence gut microbiota composition, enhancing energy expenditure and maintaining homeostasis. Low-intensity exercise may also lower intestinal transit time, reducing the opportunity for pathogens to interact with the GI mucus layer. Furthermore, physical activity increases both the quantity and diversity of gut bacteria. By positively modulating the gut microbiota, exercise might help prevent chronic migraine development instead of only relieving symptoms in affected individuals (Rojas-Valverde et al. 2023).

- *Weight reduction in migraineurs*

Obesity is potentially associated with a higher occurrence of migraines. It may also be connected to more severe migraine attacks and greater disability, particularly in women of reproductive age. Factors such as sympathetic dysregulation, adipose tissue activity, inflammatory markers like C-reactive protein and CGRP, and other neurotransmitters and neuropeptides related to hypothalamic function can all affect the link between obesity and migraine. Weight loss could help reduce both how often migraines happen and their severity (Kristoffersen et al. 2020).

- *Management of stress*

Stress and anxiety frequently occur alongside severe migraines and gastrointestinal conditions. These conditions are complex, and psychological stress can alter gut microbiota, possibly worsening migraines. Chronic stress disrupts the gut microbiome, causing dysbiosis, which can heighten inflammation and pain sensitivity (Gao et al. 2018) .

The gut-brain axis plays a role in influencing how migraines are affected by stress. When the hypothalamic-pituitary-adrenal (HPA) axis is activated, cortisol levels increase, which can alter neurotransmitter balance and inflammatory markers linked to migraine episodes (Arzani et al. 2020).

Effective stress management is essential for people with migraines. Interventions such as CBT, mindfulness meditation, and exercise have demonstrated benefits in reducing stress and enhancing mood. Incorporating these strategies into a comprehensive program allows healthcare providers to target both psychological and physiological factors, ultimately improving the quality of life for migraine sufferers (Pardos-Gascón et al. 2021).

- *Behavioral treatment*,* lifestyle modification*

Patients are strongly advised to utilize behavioral treatments such as biofeedback, to promote relaxation, mindfulness practices to reduce stress, cognitive therapy, muscle relaxation methods (since tension and relaxation cannot occur simultaneously), and desensitization through visualizing anxiety triggers(Seng and Lipton 2022). (Haghdoost and Togha 2022). Furthermore, acquiring skills to reshape unhelpful thought patterns, encouraging relaxation via diaphragmatic breathing, boosting patients’ self-confidence, instructing problem-solving techniques, and incorporating methods such as yoga, prayer, music therapy, and meditation can all be advantageous (Desai et al. 2015).

- *Probiotics and prebiotics*

Gut dysbiosis, or an imbalance of intestinal microflora, is a key factor in the development of migraines. It can lead to the release of gut-derived inflammatory cytokines like tumor necrosis factor (TNF)-α, which can affect vagus nerve activity, along with reduced production of short-chain fatty acids (SCFAs). Additionally, low microflora levels may allow E. faecalis to overgrow, increasing tyramine production and potentially triggering migraines. Therefore, dietary strategies that modulate the gut microbiota and the gut-brain axis offer a promising new adjunct therapy for migraine management, complementing pharmaceutical options. (Parohan et al. 2020; Di Lauro et al. 2023; Tirani et al. 2024) .

Probiotics are “living microorganisms” that promote human health when consumed in adequate amounts (Reiter et al. 1998); Sanders et al. 2018). Lactobacilli and Bifidobacterium are the main probiotic genera, with their strains and species affecting their functions. By restoring balance to the gut microbiota, they can alter chronic migraine symptoms. While the precise mechanism isn’t fully understood, it may include lowering proinflammatory cytokines, reducing inflammation through NF-κB pathway suppression, enhancing SCFA production, and strengthening gut epithelial integrity (Parohan et al. 2020; Di Lauro et al. 2023; Tirani et al. 2024).

A randomized double-blind controlled trial examined the effects of a multispecies probiotic supplement on migraine features and inflammatory markers. The study suggests that probiotic supplementation may decrease the frequency and severity of migraine attacks, especially in chronic migraine patients, although it does not significantly change inflammatory marker levels. Additionally, the probiotics help prevent migraine onset by controlling tyramine levels through reducing pathogenic bacterial populations (Martami et al. 2019) (Table 2).

**Table 2:** Summary of clinical trials on probiotics in migraine management

Study (Year)	Design	Sample size	Strain(s) used	Key outcomes (Effect Size)
Martami et al. (2019)	RCT	79	14-strain mix	↓ Migraine frequency (d = 0.8, *p* < 0.01)
Parohan et al. (2020)	Meta-analysis	6 RCTs	Mixed strains	↓ Attack frequency (SMD = -0.67, 95% CI: -1.23 to -0.11)
Tirani et al. (2024)	RCT	60	Lactobacillus + Bifidobacterium	↓ Severity (VAS: -2.1 ± 0.3, *p* = 0.02)

Meta-analyses show moderate effect sizes for probiotics in reducing migraine frequency (SMD = -0.67), although heterogeneity exists due to strain specificity and intervention duration (Parohan et al. 2020). For example, multispecies formulations (e.g., 14-strain mix) demonstrate more potent effects (d = 0.8) compared to single-strain trials, likely due to synergistic modulation of gut ecology (Martami et al. 2019).

Probiotics may assist if a leaky gut (permeable intestine) contributes to migraines. Live cultures such as Saccharomyces boulardii can compete with and displace harmful microbes from binding to and penetrating the intestinal wall, which helps reduce permeability. Consequently, they support the integrity of the intestinal barrier, may alleviate gastric stasis—a common GI issue in migraine sufferers—and can accelerate stomach emptying (Martami et al. 2019).

Undigested compounds called prebiotics can selectively promote the growth or activity of gut microflora. Their capacity to restore gut eubiosis reduces migraine attack frequency. Research highlights the role of the main prebiotics, fructooligosaccharides (FOSs) and galactooligosaccharides (GOSs), in potentially reducing neuroinflammation. Regarding N-methyl-D-aspartic acid (NMDA) receptors and brain neurotrophic factors, GOSs and FOSs seem to improve synaptic plasticity and brain function. Since NMDA receptors are susceptible to inflammation, neurogenic inflammation in the trigeminovascular nociceptive system is a key contributor to migraines (Di Lauro et al. 2023).

Interestingly, although triptans and CGRP inhibitors are still the primary treatments for immediate relief, dietary strategies such as the ketogenic diet demonstrate similar effectiveness to propranolol in decreasing monthly migraine days (Δ = -3.2 vs -3.5 days) (Valente et al. 2022). Probiotics could improve the effectiveness of amitriptyline by 30% in patients with chronic migraines, indicating potential benefit from combined therapy (Tirani et al. 2024).

- *The impact of diet and nutrition on migraine severity*

Many foods and drinks are known migraine triggers, including dairy, chocolate, caffeine, and alcohol, which affect 27% of migraine sufferers. Conversely, specific vitamins and minerals like thiamine, magnesium, and riboflavin have shown potential in reducing headache pain (Zaeem et al. 2016). Caffeine can both alleviate and cause headaches, depending on factors like dosage, timing, and individual metabolism. Additionally, withdrawal from long-term caffeine use may result in headaches (Nowaczewska et al. 2020).

Multiple studies highlight the importance of diet in health, emphasizing the need for regular meal patterns and identifying specific food triggers. Managing these dietary factors can help patients achieve better well-being and more effective symptom control in conditions like migraine and gastrointestinal diseases (Tai et al. 2018; Kim et al. 2022).

Migraines are a common and debilitating neurological condition that can be difficult to control with medication. Non-drug approaches, like dietary changes, have been explored as methods to prevent migraines (Gazerani 2020).

- Low-fat diets may affect migraine severity depending on gut microbiota and metabolites. High-fat ingredients produce prostaglandins like E1, which can trigger headaches by increasing inflammation (Roldán-Ruiz et al. 2025). In addition, High-fat diets increase platelet aggregation, raising serotonin levels and prostaglandin production, which contribute to more severe migraines. Conversely, low-fat diets reduce blood free fatty acids and lipids, potentially limiting pro-inflammatory prostaglandins (Heinritz et al. 2016). More importantly, a low-fat diet can favorably alter gut microbiota composition. Beneficial bacteria that produce metabolites like SCFAs, such as butyrate, tend to be more abundant on lower-fat diets. These SCFAs have anti-inflammatory effects, can modulate immune responses, improve gut barrier function, and may reduce systemic inflammation associated with migraines (Markowiak-Kopeć and Śliżewska 2020).

Experiments suggest a homeostatic gut microbiome may influence pain pathways, inflammation, and migraine severity through bacterial metabolites. A low-fat diet improves microbiome balance, reducing migraine frequency and severity, but the relationship among diet, microbiota, and migraine is complex (Jiang et al. 2018). Future research will explore how dietary changes modify gut microbiota and metabolites, providing new migraine therapy targets.

- Omega-3 fatty acid supplementation: Evidence suggests that dietary oils rich in omega-3s can help lessen migraine symptoms. Polyunsaturated fatty acids (PUFAs), particularly long-chain omega-3s like eicosapentaenoic acid (EPA) and docosahexaenoic acid (DHA) found in cold-water oily fish and over-the-counter fish oil supplements, have been shown to positively impact inflammatory processes. Omega-3s can lower levels of pro-inflammatory cytokines and C-reactive protein (CRP), both of which play a role in migraine development (Maghsoumi-Norouzabad et al. 2017).

Additionally, omega-3 supplements have been associated with beneficial alterations in gut microbiota composition. By encouraging the growth of beneficial bacteria, omega-3 PUFAs can boost the production of SCFAs like butyrate, which has anti-inflammatory properties and may support improved gut barrier function. A balanced gut microbiome helps maintain a healthy immune response, with its metabolites shown to affect pain pathways and inflammatory mediators, thereby potentially reducing migraine severity (Soares et al. 2017).

These novel nutritional therapies serve as an additional option alongside pharmacological treatments for migraines. By targeting the gut microbiota and the gut-brain axis, omega-3 fatty acids present a promising opportunity to improve migraine management and highlight the therapeutic potential of nutritional approaches (Djalali et al. 2023).

- Vitamin supplementation: There are many dietary supplements available for use as supplementary migraine treatments. The vitamin B family significantly helps prevent migraines. Meat, sprouts, rice bran, and eggs are the primary sources of vitamin B1. Conversely, leafy vegetables, milk, eggs, and yeast are good sources of vitamin B2. Vitamin B6 is abundant in sweet potatoes, fish, and other foods (Nematgorgani et al. 2022).

A previous study indicated that vitamin supplementation can benefit gut microbiota composition. Certain B vitamins have been found to promote the growth of beneficial gut microbes that produce SCFAs like butyrate, which may help reduce systemic inflammation involved in migraine development. Thus, vitamin supplements can serve as an additional therapy alongside standard treatments (Ariyanfar et al. 2022).

- The ketogenic diet (KD) is a low-carb, high-fat weight-loss regimen also used to treat epilepsy. Interestingly, it might also help with migraines. A study involving 33 migraine patients treated with KD found that neurological assessments including headache frequency, medical history, and medication use over the previous month showed that five patients (65.2%) experienced at least a 50% reduction in both headache duration and frequency, decreasing from an average of 12.5 to 6.7. This improvement was observed after three months on the diet (Valente et al. 2022). Moreover, KD alters gut microbiota by promoting beneficial bacteria that metabolize fats and suppress pro-inflammatory bacteria. This shift increases health-promoting metabolites like butyrate, which maintains gut barrier integrity and reduces systemic inflammation, thereby decreasing migraine severity. (Barbanti et al. 2017; Lovati et al. 2022).
- *The Mediterranean ketogenic diet* Consuming a diet rich in antioxidant and anti-inflammatory compounds like fruits, vegetables, whole grains, legumes, nuts, and healthy fats such as olive oil, and reducing red meat and processed food intake resulted in significant reductions in the intensity, severity, and frequency of migraine pain (Gazerani 2020). A previous study suggested that combining the anti-inflammatory benefits of the Mediterranean diet with the metabolic effects of ketosis could be an effective treatment for migraine (Bakırhan et al. 2021). A cross-sectional study indicated that following the Mediterranean diet is linked to fewer, shorter, and less severe migraine attacks (Arab et al. 2021). Overall, combining Mediterranean dietary principles with other diets like the ketogenic diet is a promising future approach for enhancing migraine management and boosting patient quality of life.

### Natural products used in migraine management

**Fig. 5:**
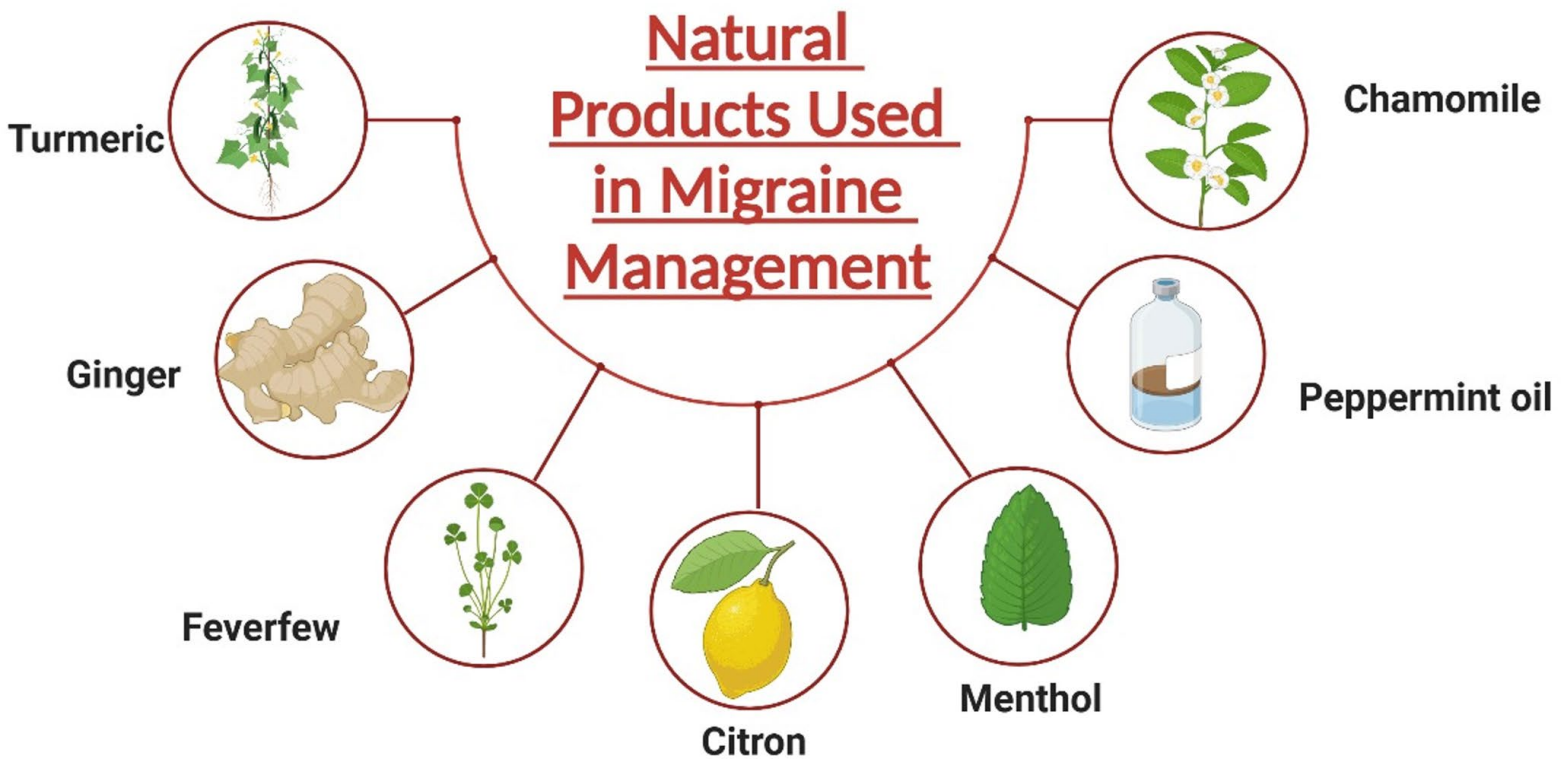
Natural products used in migraine management

The use of herbal supplements and plants as complementary options alongside conventional pharmaceuticals for migraine treatment is steadily increasing. (Fig. 5). Turmeric, which contains the active ingredient curcumin, has anti-inflammatory properties that may help soothe headaches. Regular consumption of turmeric has been linked to a reduction in migraine attacks, possibly by balancing gut bacteria, increasing *Bifidobacterium* levels, decreasing *Clostridium*, and lowering inflammation originating from the gut (Kaur et al. 2021).

Ginger is a widely used herb known for its anti-inflammatory effects and has been studied for migraine treatment. It is generally safe for most people and offers gut health benefits, which can influence Lactobacillus populations. These bacteria are linked to decreased migraine frequency through serotonin production (Andrade 2021).

Feverfew migraine has been used historically to treat migraines and headaches by reducing attack frequency and symptoms like nausea and vomiting. Some studies suggest its effectiveness is due to its influence on gut microbiota (Ernst and Pittler 2000; Pittler and Ernst 2004). Although evidence is limited regarding butterbur (Petasites hybridus) for migraine prevention, growing interest focuses on its potential impact on gut health. Additionally, citron (Citrus medica) and coriander have shown early promise in preventing migraines, possibly through mechanisms involving gut microbiota (Lopresti et al. 2020).

Menthol and peppermint oil have also proven helpful in treating acute migraines and, according to some studies, may benefit gut health. Chamomile shows promise as a treatment for acute migraines in early research, thanks to its sedative properties and its ability to support gut health (Lopresti et al. 2020). Uncovering how natural molecules interact with gut microbiota could open up new therapeutic options for managing migraine via the gut-brain axis.

### Nutraceuticals as a treatment for migraine

Magnesium is a mineral that effectively reduces the frequency of migraines and is often used as a preventative measure. Vitamin B2, or riboflavin, has been shown to stop migraine attacks when taken at high doses. Additionally, regular consumption of Co-enzyme Q10 (CoQ10), an antioxidant, has been found to lower both the frequency and severity of migraines (Kaur et al. 2021).

### Novel routes of drug administration of for migraine treatment

**Fig. 6:**
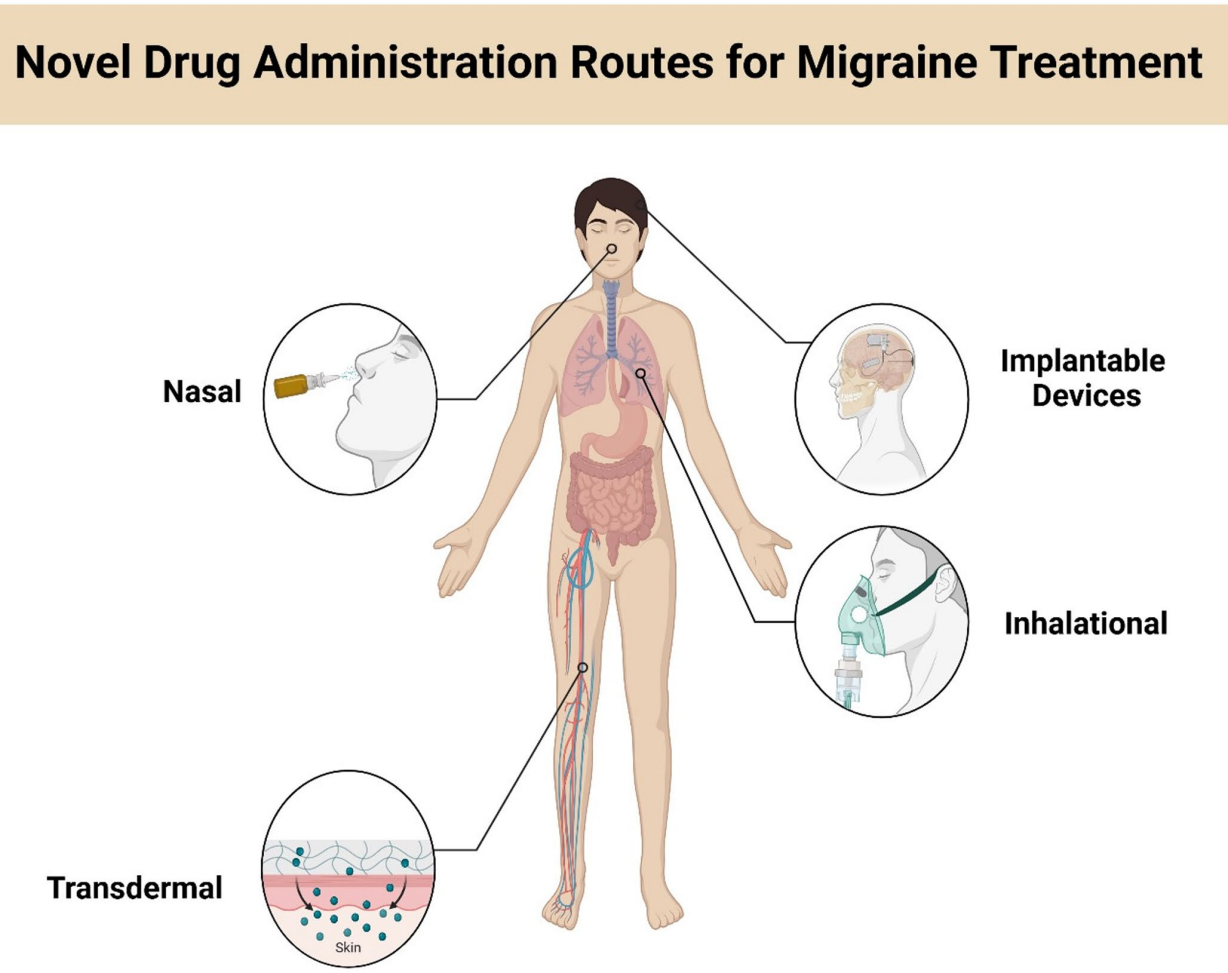
Novel routes of administration of a drug for migraine treatment

Drug delivery methods improve treatment effectiveness and reduce side effects for brain disorders. (Fig. 6). The blood-brain barrier (BBB) is an essential part of the central nervous system that controls the entry of blood-borne molecules into the brain. Erodible polymer nanoparticles (NPs) are preferred drug delivery vehicles because they are biodegradable and easy to eliminate. These NPs, which are submicron colloidal carriers measuring 10 to 100 nm in diameter, provide benefits such as a high surface-to-volume ratio, targeted delivery, and controlled release, thereby reducing the risk of dose dumping (El-Say and El-Sawy 2017). These features may improve patient adherence by decreasing the frequency and dosage of medication. Here are examples of nanoparticle-based drug delivery systems (NDDS) used for migraine treatment:

Intranasal delivery has seen the rise of chitosan nanoparticles (CSNPs) as a promising technology over the past decade. In 2005, researchers developed cationic nanoparticle complexes loaded with ammonium glycyrrhizinate and chitosan-TT (Wu et al. 2005). Further research in 2015 demonstrated that CSNPs could successfully deliver sumatriptan succinate through the intranasal route for migraine relief (Hansraj et al. 2015). AVP-825, a low-dose dry nasal powder of sumatriptan (22 mg), is intended for rapid relief of migraines, minimizing deposition in the oropharynx and lungs. This formulation provides improved stability, increased adhesion to the nasal mucosa, and contains fewer preservatives (Silberstein 2017).

Phase II and III clinical trials indicated that AVP-825 can effectively reduce early migraine symptoms with few triptan-related side effects. None of the three controlled studies reported significant adverse effects, and safety data from placebo groups matched those from efficacy studies like Phase III COMPASS. The systemic side effects of AVP-825 are similar to those of 100 mg oral sumatriptan (Cady 2015; Tepper et al. 2015).

Transdermal Iontophoretic delivery of sumatriptan offers a promising, non-invasive alternative for patients with absorption issues or severe side effects from triptans. It provides quick, steady plasma concentrations for migraine treatment. The pharmacokinetic study confirmed rapid, consistent drug delivery through the skin (Vikelis et al. 2012).

Implantable Devices: Occipital nerve stimulation (ONS) has shown long-term efficacy and tolerability in treating intractable chronic migraine. Clinical trials showed patients experienced substantial pain relief, with a 4.9 ± 2.0 point decrease on the Visual Analogue Scale (VAS). These results persisted over 7 years, indicating ONS may provide sustained relief. Some patients reported complete migraine cessation, while others noted pain reduction. Success depends on patient selection, precise programming, surgical technique, psychological evaluations, and occipital nerve blocks. Further research is needed to understand its mechanisms fully. (Young 2014). Overall, ONS could be a viable long-term treatment for patients with chronic migraines, as it helps decrease pain signals by influencing thalamic activity and lowering CGRP release in the trigeminal nucleus, thereby breaking the cycle of peripheral and central sensitization. Additionally, ONS may significantly enhance quality of life.

### Patient perspectives and their impact on quality of life

Many studies have concentrated on how migraines affect patients’ quality of life. People with migraines want to be involved in their treatment decisions, stressing the importance of shared decision-making, personalized care plans, and trust with healthcare providers. They value a holistic approach that addresses more than just headache symptoms. Patients appreciate open dialogue, feeling recognized and validated, along with mutual respect in their relationships with providers. Research shows that migraine sufferers often face stigma and misunderstanding, leading to feelings of isolation and challenges in obtaining appropriate care (Tana et al. 2024).

Patients often highlight the significant impact of migraines on their family life, work, and social relationships, as well as difficulties with medical care. The stigma associated with migraines mainly results from a lack of awareness and their invisible symptoms, emphasizing the need to change how migraines are discussed to combat discrimination and stigma. Moreover, alternative treatments like acupuncture have demonstrated benefits for mood, quality of life, and overall well-being in migraine sufferers. Acupuncture not only reduces pain intensity but also enhances the Migraine-Specific Quality of Life (MSQ) score, indicating an improved quality of life. Its capacity to target both physical and emotional facets makes it a comprehensive treatment option that goes beyond simple pain relief (Battista et al. 2023).

### Clinicians should consider the following strategies for migraine management

- Recommend probiotic strains with established effectiveness, such as Lactobacillus rhamnosus GG and Bifidobacterium longum, for 8–12 weeks (Tirani et al. 2024).
- Recommend a Mediterranean-Ketogenic hybrid diet to lower inflammation and enhance gut barrier health (Lovati et al. 2022).
- Screen for comorbid gastrointestinal disorders such as IBS and H. pylori, and treat gut dysbiosis in addition to migraine-specific therapies (Arzani et al. 2020).
- Monitor drug-diet interactions (e.g., triptans with tyramine-rich foods) (Nedergaard et al. 2024).

### Future directions in migraine-gut axis research: unveiling new therapeutic targets and strategies

Calcitonin gene-related peptide (CGRP) and pituitary adenylate cyclase-activating polypeptide (PACAP) are neuropeptides released during migraine and cluster headache episodes, acting as potent vasodilators that cause migraine-like symptoms. Recent advances have led to the development of new treatments that target the CGRP signaling pathway for migraine management and prevention. These include monoclonal antibodies (mAbs) aimed at the CGRP ligand or receptor, as well as CGRP receptor inhibitors. Currently, four mAb products are available. The three humanized monoclonal antibodies targeting the CGRP ligand are eptinezumab, fremanezumab, and galcanezumab, while erenumab is a fully human monoclonal antibody that targets the main CGRP receptor. Despite slight differences in their binding affinities, these four drugs show similar effectiveness, tolerability, and cause minimal side effects (Cohen et al. 2022) (Dominguez-Moreno et al. 2022).

Compared to small-molecule CGRP antagonists called gepants, these mAbs provide a longer half-life, lower risk of drug interactions, and greater target specificity. Their mode of action depends on their specific targets and how they distribute in the body. Unlike small molecules, mAbs cannot cross the blood-brain barrier. Still, they can reach the dura mater and sensory ganglia such as the vagus, sphenopalatine, and trigeminal ganglia, where they likely act. CGRP mAbs modify downstream signaling pathways and block CGRP activity (Takizawa et al. 2023).

In vitro studies indicate that fenamenezumab and eptinezumab have a stronger affinity for the CGRP ligand compared to galcanezumab and erenumab, which bind reversibly to their targets. Rat studies demonstrate that CGRP monoclonal antibodies (mAbs) can block neurogenic vasodilation without impacting arterial blood pressure or heart rate. Ideally, a CGRP blocker remains inactive when CGRP is absent. In a rat model of cortical spreading depression, CGRP mAbs were specifically effective in reducing Aδ-fiber reactivity, while C-fibers were unaffected (Cohen et al. 2022).

Research has shown that NADPH oxidase 2 (NOX2) in nitroglycerin (NTG) plays a role in elevating oxidative stress and inflammation through the gut-brain axis during the early phase of a migraine. Additionally, the interaction between NOX2 and Nuclear Factor Erythroid 2-related Factor 2 (Nrf2) was explored, indicating a promising method to lower NOX2 levels and reduce oxidative stress and inflammation in both neurological and non-neurological conditions. Restoring the Nrf2 and NOX2 balance can inhibit NF-κB and NLRP3 inflammasome activation, preventing neuronal damage and decreasing glial reactivity. In the colon, increasing Nrf2 expression and decreasing NOX2 resulted in less tissue damage, fewer mast cells, and lower release of pro-inflammatory cytokines such as IL-1β and TNF-α (Tastan et al. 2022). The findings imply that activating Nrf2 could be beneficial in managing migraine symptoms and related comorbidities. This highlights the Nrf2/NOX2 axis as a promising therapeutic target for migraines and associated gastrointestinal conditions.

Tempol has been demonstrated to reduce clinical symptoms of migraine and abdominal pain depending on the dose. The Nrf2 inducer tempol was used to further investigate the role of the Nrf2/NOX2 axis in migraine and associated gastrointestinal problems (Silva et al. 2020). In a mouse model study, nitroglycerin (NTG) was used to induce migraines. In contrast, different doses of tempol (10 mg/kg, 30 mg/kg, and 100 mg/kg) were administered. Researchers employed behavioral tests such as the Von Frey test, tail-flick test, and light/dark test to evaluate migraine symptoms. Additionally, histological analyses of brain and colon tissues were performed to assess tissue damage. The findings suggest that tempol might significantly reduce tissue damage and help alleviate migraine symptoms (Ardizzone et al. 2024).

Fecal microbiota transplantation (FMT) is gaining recognition as a promising approach for treating migraines by altering gut bacteria and their metabolic products, especially short-chain fatty acids (SCFAs) such as propionate and butyrate (Halaweish et al. 2022). FMT rebalances gut microbes such as Bacteroides, Roseburia, and Faecalibacterium species, which produce beneficial compounds. This process helps modulate immune responses, including T-cell activity and anti-inflammatory signals like IL-10, while also decreasing neural inflammation (Vinolo et al. 2011). By improving gut-derived metabolites and increasing microbial diversity, FMT targets disruptions in the gut-brain axis associated with migraine development. This provides a new approach for reducing chronic pain through therapies focused on the microbiome (Pandey and Tiwari 2024).

## Limitations

This review focuses primarily on gut-brain axis mechanisms and adjunctive therapeutic strategies rather than providing exhaustive systematic reviews or meta-analyses of all acute and preventive migraine therapies. The narrative approach, while comprehensive in scope, may not capture all nuances that would emerge from formal systematic review methodology with standardized quality assessment tools. Additionally, recent therapeutic developments were included through targeted literature searches rather than comprehensive systematic database screening, which may have introduced selection bias toward more prominently published studies.

While this review presents evidence for gut-brain axis involvement in migraine pathophysiology, many of the proposed mechanisms remain incompletely understood. The relationship between specific microbiome alterations and migraine severity remains largely associative rather than demonstrating clear causality. Most human studies are cross-sectional or short-term interventional trials that cannot establish whether microbiome changes are cause, consequence, or merely correlative with migraine patterns.

The neuroinflammatory pathways connecting gut dysbiosis to trigeminal sensitization require further elucidation. While preclinical models suggest roles for bacterial metabolites (particularly short-chain fatty acids), lipopolysaccharide translocation, and cytokine signaling cascades, the translation of these mechanisms to human migraine pathophysiology remains speculative in many instances.

Regarding acute treatment approaches, analgesic efficacy may be substantially lower than triptan or gepant-class agents in severe migraine attacks, particularly those associated with high disability scores or rapid pain escalation. The safety profiles of common analgesics vary considerably based on individual patient risk factors, including gastrointestinal history, cardiovascular comorbidities, renal function, and concurrent medications, making individualized risk-benefit assessments essential but complex.

The medication overuse headache risk associated with frequent analgesic use represents a significant clinical limitation, particularly for patients with high-frequency episodic migraine who may benefit from frequent acute treatment but risk progression to chronic daily headache patterns. The threshold of â‰¥15 days per month for NSAIDs represents population-level guidance that may not apply uniformly to all individuals.

The development of personalized treatment approaches based on individual microbiome profiles, genetic polymorphisms affecting drug metabolism, or biomarkers predicting treatment response remains largely theoretical. Current evidence does not support routine microbiome analysis for clinical decision-making in migraine management. However, this may change as research methodologies and our understanding of gut-brain mechanisms continue to evolve.

## Conclusion

The bidirectional relationship between the gut-brain axis represents a fundamental paradigm shift in migraine understanding. It reveals that gastrointestinal health directly influences trigeminal nociception through neural, immune, endocrine, and metabolic pathways. Gut microbiota alterations, intestinal barrier dysfunction, and neuroinflammation create pathophysiological cascades affecting migraine susceptibility and treatment response. In contemporary clinical practice, CGRP-pathway preventive therapies, including monoclonal antibodies (erenumab, eptinezumab, fremanezumab, galcanezumab) and gepants (atogepant, rimegepant, ubrogepant, zavegepant) currently provide the most consistent evidence-based benefits, with the 2024 American Headache Society consensus statement recommending CGRP mAbs as first-line therapy based on superior efficacy (50–60% achieving ≥ 50% reduction in monthly migraine days) and tolerability compared to traditional preventives, while external neuromodulation devices, including Cefaly and Relivion, offer valuable non-pharmacological alternatives. Common analgesics remain essential for acute care, with ibuprofen 400–800 mg providing superior efficacy and flurbiprofen demonstrating both acute and prophylactic benefits. However, the 15-day monthly threshold necessitates careful monitoring, given recent evidence that chronic NSAID use leads to intestinal hyperpermeability and elevated lipopolysaccharide levels. Adjunctive gut-brain axis interventions, including ketogenic dietary approaches, probiotic supplementation with strains such as Lactobacillus helveticus and Bifidobacterium longum, and novel delivery systems like iontophoretic patches (Zelrix™) and breath-powered formulations (AVP-825), show moderate-quality evidence as complementary strategies. Understanding these interconnected mechanisms enables healthcare providers to implement multimodal approaches integrating evidence-based pharmacological treatments with gut-brain axis considerations, representing a transformational advancement in personalized migraine management for millions of affected individuals.

## Data Availability

No datasets were generated or analysed during the current study.

## Abbreviations

5: HT-serotonin (5-hydroxytryptamine)
ANS: Autonomic nervous system
BBB: Blood-brain barrier
CGRP: Calcitonin gene-related peptide
CM: Chronic migraine
CNS: Central nervous system
DHE: Dihydroergotamine
DGBI: Disorders of gut-brain interaction
EM: Episodic migraine
ENS: Enteric nervous system
FOS: Fructooligosaccharides
GABA: Gamma-aminobutyric acid
GI: Gastrointestinal
GID: Gastrointestinal disorders
GIT: Gastrointestinal tract
GOS: Galactooligosaccharides
H. pylori (HPI): Helicobacter pylori infection
HPA: Hypothalamus pituitary adrenal
IBD: Inflammatory bowel disease
IBS: Irritable bowel syndrome
IL: 1β-interleukin 1 beta
IL: 6-Interleukin 6
LPS: Lipopolysaccharides
MNP: Microneedle patch
NF: κB-nuclear factor kappa B
NMDA: N-methyl-D-aspartate
NOX2: NADPH oxidase 2
Nrf2: Nuclear factor erythroid 2-related factor 2
NTS: Nucleus tractus solitarius
PACAP: Pituitary adenylate cyclase-activating polypeptide
SCFAs: Short-chain fatty acids
TNF: α-tumor necrosis factor alpha
VAS: Visual analogue scale
